# Soluble urokinase plasminogen activator receptor (suPAR) as an early predictor of severe respiratory failure in patients with COVID-19 pneumonia

**DOI:** 10.1186/s13054-020-02897-4

**Published:** 2020-04-30

**Authors:** Nikoletta Rovina, Karolina Akinosoglou, Jesper Eugen-Olsen, Salim Hayek, Jochen Reiser, Evangelos J. Giamarellos-Bourboulis

**Affiliations:** 1 1st Department of Pulmonary Medicine and Intensive Care Unit, National and Kapodistrian University of Athens, Medical School, 115 27 Athens, Greece; 2 Department of Internal Medicine, University of Patras, Medical School, 265 04 Rio, Greece; 3 Clinical Research Centre, Copenhagen University Hospital, Hvidovre, Denmark; 4 Department of Medicine, University of Michigan, Ann Arbor, MI 48103 USA; 5 Department of Medicine, Rush University Medical Center, 1717 West Congress Parkway, Chicago, IL 60612 USA; 6 4th Department of Internal Medicine, National and Kapodistrian University of Athens, Medical School, 124 62 Athens, Greece; 7 4th Department of Internal Medicine, ATTIKON University Hospital, 1 Rimini Street, 12462 Athens, Greece

**Keywords:** COVID-19, suPAR, Respiratory failure, Prognosis

As of April 1, 2020, 885,689 cases of infections by the novel coronavirus SARS-CoV-2 (COVID-19) have been recorded worldwide; 44,217 of them have died (https://www.worldometers.info/coronavirus). At the beginning of the illness, patients may experience low-degree fever or flu-like symptoms, but suddenly, severe respiratory failure (SRF) emerges [1]. Increased circulating levels of D-dimers [1, 2] suggest endothelial activation. Urokinase plasminogen activator receptor (uPAR) that is bound on the endothelium may be cleaved early during the disease course leading to an increase of its soluble counterpart, namely suPAR [3]. If this holds true, then suPAR may be used as an early predictor of the risk of SRF.

The Hellenic Sepsis Study Group (HSSG, www.sepsis.gr) is collecting clinical information and serum samples within the first 24 h of admission from patients with infections and at least two signs of the systemic inflammatory response syndrome. Since March 1, 2020, 57 patients with community-acquired pneumonia and molecular documentation of SARS-CoV-2 in respiratory secretions were enrolled. Patients were followed up daily for 14 days; the development of SRF defined as PO_2_/FiO_2_ ratio less than 150 requiring mechanical ventilation (MV) or continuous positive airway pressure treatment (CPAP) was recorded. suPAR was measured by an enzyme immunoassay in duplicate (suPARnostic™, ViroGates, Lyngby, Denmark); the lower detection limit was 1.1 ng/ml. Measurements were performed and reported by one technician who was blinded to clinical information. The study endpoint was the prognostic performance of suPAR admission levels for the development of SRF within 14 days. Measured levels were compared to those collected from 15 patients with COVID-19 from the emergency department (ED) of Rush University Medical Center.

Thirty-four (59.6%) patients were male and 23 (40.1%) female; the mean ± SD age was 64.0 ± 10.3 years, and the Charlson’s comorbidity index was 2.70 ± 1.80. The mean ± SD admission total neutrophil count was 4414.1 ± 2526.5/mm^3^; the total lymphocyte count was 1149.1 ± 1131.4/mm^3^; the C-reactive protein was 73.1 ± 76.4 mg/l. Admission levels of suPAR were significantly greater among patients who eventually developed SRF (Fig. 1a). Circulating levels of suPAR were of the same range as those of the US cohort (Fig. 1b). Receiver operator characteristics curve analysis identified levels ≥ 6 ng/ml as the best predictor for SRF. At that cutoff point, the sensitivity, specificity, positive predictive value, and negative predictive value for the prediction of SRF was 85.7%, 91.7%, 85.7%, and 91.7%, respectively. The time to SRF was much shorter among patients with suPAR ≥ 6 ng/ml (Fig. 1c). The only admission variables that were independently associated with the development of SRF were male gender and suPAR ≥ 6 ng/ml (Table 1). A positive association was found between admission suPAR and D-dimers (*r*_s_ = + 0.777, *p* < 0.0001).

**Fig. 1:**
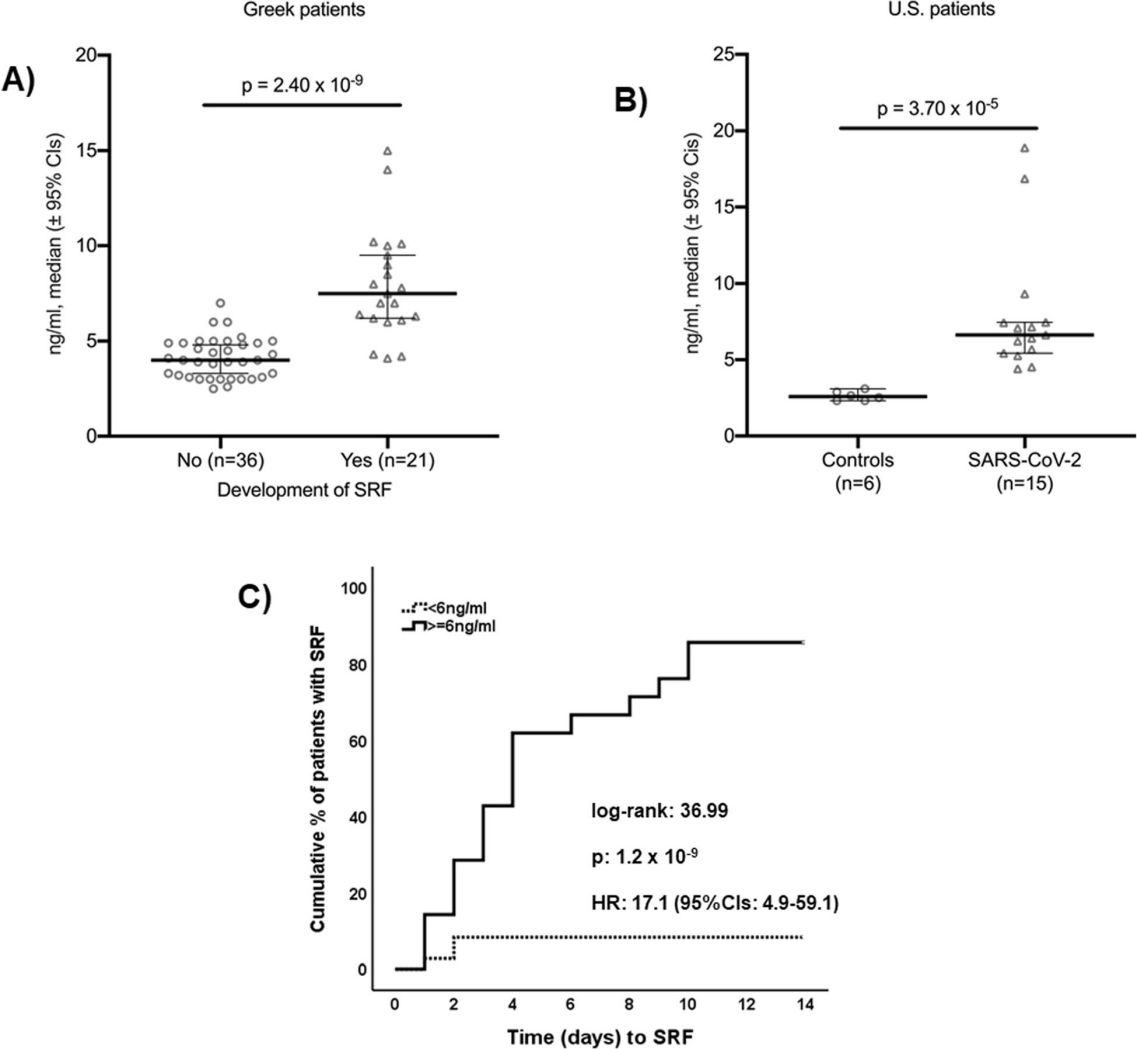
suPAR as an early predictor of the development of severe respiratory failure (SRF). **a** Admission levels of suPAR among Greek patients who eventually developed or not SRF. The *p* value of comparisons by the Mann-Whitney *U* test is provided. **b** Levels of suPAR in patients with COVID-19 and controls. The *p* value of comparisons by the Mann-Whitney *U* test is provided. **c** Time to SRF of Greek patients in relation to the admission levels of suPAR. CI, confidence interval; HR, hazard ratio

**Table 1:** Independent variables at admission associated with the development of severe respiratory failure

	No need for MV or CPAP, *n* (%)	Need for MV or CPAP, *n* (%)	Univariate analysis		Forward Cox regression analysis	
			OR (95%CIs)	*p* value	HR (95%CIs)	*p* value
Male gender	15 (41.7)	19 (90.5)	0.07 (0.02–0.37)	< 0.0001	7.80 (1.75–34.76)	0.007
CCI > 2	17 (48.6)	17 (77.3)	7.00 (2.11–24.25)	0.002		ns
suPAR ≥ 6 ng/ml	3 (8.3)	18 (85.7)	66.00 (12.05–361.35)	< 0.0001	16.43 (4.56–59.19)	< 0.0001
Neutrophils ≥ 4200/mm^3^	8 (22.2)	16 (72.2)	11.20 (3.13–40.08)	< 0.0001		ns
CRP ≥ 58 mg/l	7 (19.4)	13 (61.9)	6.73 (2.01–22.51)	0.002		ns

*CCI* Charlson’s comorbidity index, *CRP* C-reactive protein, *CI* confidence interval, *HR* hazard ratio, *OR* odds ratio

suPAR has been proposed as a biomarker for the risk of death. An analysis of the TRIAGE III trial in 4420 patients admitted at the ED in Denmark revealed that suPAR ranged between 2.6 and 4.7 ng/ml in 30-day survivors and between 6.7 and 11.8 ng/ml in 30-day non-survivors [4]. Early increase of suPAR has also been reported to be a prediction of 28-day outcome in sepsis [5]. uPAR is bound to the endothelial membrane and functions for the differential signaling between the cleaved and uncleaved forms of kininogen [3]. The positive association between D-dimers and suPAR suggest early complex kininogen and uPAR interactions at the endothelial level of early stages of COVID-19. Higher plasma levels of suPAR are predictive of and potentially causally involved in kidney disease [6] which can be a feature of severe COVID-19 infection.

Findings suggest that suPAR may early trace patients who need intensified management probably in need of anti-inflammatory treatment [6]. Whether modification of circulating suPAR is a useful therapeutic option will require further study.

## Data Availability

The datasets used and/or analyzed during the current study are available from the corresponding author upon reasonable request.

## Abbreviations

CCI: Charlson’s comorbidity index
CI: Confidence interval
COVID-19: Infection by the novel coronavirus SARS CoV-2
CPAP: Continuous positive airway pressure treatment
HR: Hazard ratio
MV: Mechanical ventilation
NPV: Negative predictive value
PPV: Positive predictive value
ROC: Receiver operating characteristic
SD: Standard deviation
SRF: Severe respiratory failure
suPAR: Soluble urokinase plasminogen activator receptor
